# Modulation of Astrocytic Mitochondrial Function by Dichloroacetate Improves Survival and Motor Performance in Inherited Amyotrophic Lateral Sclerosis

**DOI:** 10.1371/journal.pone.0034776

**Published:** 2012-04-03

**Authors:** Ernesto Miquel, Adriana Cassina, Laura Martínez-Palma, Carmen Bolatto, Emiliano Trías, Mandi Gandelman, Rafael Radi, Luis Barbeito, Patricia Cassina

**Affiliations:** 1 Departamento de Histología y Embriología, Facultad de Medicina, Universidad de la República, Montevideo, Uruguay; 2 Departamento de Bioquímica, Facultad de Medicina, Universidad de la República, Montevideo, Uruguay; 3 CEINBIO, Facultad de Medicina, Universidad de la República, Montevideo, Uruguay; 4 Instituto de Investigaciones Biológicas Clemente Estable, Montevideo, Uruguay; 5 Institut Pasteur de Montevideo, Montevideo, Uruguay; Federal University of Rio de Janeiro, Brazil

## Abstract

Mitochondrial dysfunction is one of the pathogenic mechanisms that lead to neurodegeneration in Amyotrophic Lateral Sclerosis (ALS). Astrocytes expressing the ALS-linked SOD1^G93A^ mutation display a decreased mitochondrial respiratory capacity associated to phenotypic changes that cause them to induce motor neuron death. Astrocyte-mediated toxicity can be prevented by mitochondria-targeted antioxidants, indicating a critical role of mitochondria in the neurotoxic phenotype. However, it is presently unknown whether drugs currently used to stimulate mitochondrial metabolism can also modulate ALS progression. Here, we tested the disease-modifying effect of dichloroacetate (DCA), an orphan drug that improves the functional status of mitochondria through the stimulation of the pyruvate dehydrogenase complex activity (PDH). Applied to astrocyte cultures isolated from rats expressing the SOD1^G93A^ mutation, DCA reduced phosphorylation of PDH and improved mitochondrial coupling as expressed by the respiratory control ratio (RCR). Notably, DCA completely prevented the toxicity of SOD1^G93A^ astrocytes to motor neurons in coculture conditions. Chronic administration of DCA (500 mg/L) in the drinking water of mice expressing the SOD1^G93A^ mutation increased survival by 2 weeks compared to untreated mice. Systemic DCA also normalized the reduced RCR value measured in lumbar spinal cord tissue of diseased SOD1^G93A^ mice. A remarkable effect of DCA was the improvement of grip strength performance at the end stage of the disease, which correlated with a recovery of the neuromuscular junction area in extensor digitorum longus muscles. Systemic DCA also decreased astrocyte reactivity and prevented motor neuron loss in SOD1^G93A^ mice. Taken together, our results indicate that improvement of the mitochondrial redox status by DCA leads to a disease-modifying effect, further supporting the therapeutic potential of mitochondria-targeted drugs in ALS.

## Introduction

ALS is a fatal paralytic neurodegenerative disease characterized by motor neuron loss, which leads to death within 3–5 years of diagnosis. No therapy is available other than riluzole, which extends the lifespan of patients by only 3–6 months [1]. Mitochondrial dysfunction can contribute to motor neuron degeneration in ALS and description of mitochondrial alterations have been fully documented in the spinal cord and the muscle from patients and animal models linked to SOD mutations (for review see [2], [3]). Astrocytes from rats expressing SOD1^G93A^ display reduced mitochondrial ability to synthesize ATP and produce increased levels of nitric oxide [4], superoxide and peroxynitrite [5] and accordingly, these effects can be reverted with mitochondria-targeted antioxidants [5]. Interestingly, mitochondrial dysfunction in astrocytes is associated to neurotoxic phenotypic changes that reduce motor neuron survival [5].

Astrocytes surrounding motor neurons are known to modulate ALS progression. Indeed, analyses of chimeric mice composed of mixtures of normal and mutated SOD1–expressing cells have offered evidence that motor neuron death is non–cell-autonomous [6]. Restricted mutated SOD1 expression in astrocytes is not sufficient for disease development [7]. However, selective reduction of mutated SOD1 in astrocytes increases disease duration after onset as determined by mating mice expressing mutated SOD1 transgenes flanked by lox sites to mice carrying a Cre-encoding sequence under control of the promoter from GFAP [8]. This is accompanied by delayed microglial activation, in accordance with studies using the same technology for microglia [9]. Damage induced by mutated SOD1 in astrocytes determines a phenotype that is neurotoxic for motor neurons in culture and may account for the role of astrocytes in disease progression [4], [10], [11]. Indeed, the recent isolation of astrocytes with aberrant phenotype (referred to as “AbA cells”) from primary spinal cord cultures of symptomatic SOD1^G93A^ rats with unprecedented proliferative and neurotoxic capacity [12] further supports a role for astrocytes in ALS progression. It remains to be determined whether the neurotoxic phenotype of SOD1^G93A^-expressing astrocytes may be reverted by the improvement of mitochondrial metabolism and in turn slow disease progression.

**Figure 1:**
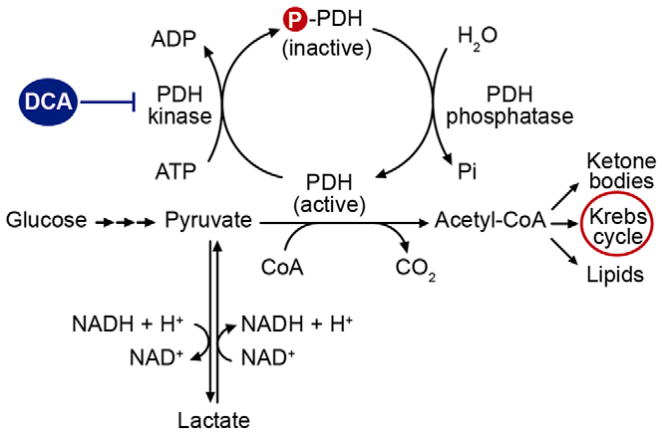
Site of action of dichloroacetate. DCA inhibits the mitochondrial enzyme PDH kinase, thereby maintaining the PDH complex in its unphosphorylated catalytically active state and facilitating the aerobic oxidation of glucose.

The organohalide dichloroacetate (DCA) is a well-characterized inhibitor of the protein kinase of the pyruvate dehydrogenase (PDH) [13]. PDH, located in the mitochondrial matrix, in its active unphosphorylated state mediates acetyl coenzyme-A formation from pyruvate, which feeds the electron transport chain responsible for ATP synthesis and oxygen consumption. Phosphorylation of PDH by PDH kinase (PDK) generates its inactive phosphorylated state. DCA-mediated inhibition of PDK renders most of PDH in the active form and then pyruvate metabolism switches towards glucose oxidation to CO_2_ in the mitochondria (Fig. 1). Another mechanism by which DCA may favor PDH activity is to decrease degradation of the E1 alpha subunit of the complex. It has been claimed that changes in E1 alpha subunit phosphorylation could affect susceptibility to proteases that may lead to an increase in the amount of the total enzyme [14].

In the central nervous system, DCA enhances glucose and lactate oxidation to CO_2_ and reduces lactate release mainly in astrocytes compared to having almost no effects on neurons, which supports the compartmentalization of glucose metabolism between astroglia and neurons [15]. The fraction of total PDH in the inactive phosphorylated form is normally greater in astroglia than in neurons, a situation that favors lactate export from astroglia to neurons but it can be modulated by DCA [15]. DCA administration *in vivo* activates brain PDH activity [16], indicating that it crosses the blood-brain barrier, and DCA is currently used clinically to lower elevated lactate levels in cerebrospinal fluid of patients with mitochondrial disorders [17], [18]. However, it is unknown whether DCA may offer benefits in neurological disorders associated to mitochondrial dysfunction. Specifically, it is not known whether DCA may prevent the *in vivo* and *in vitro* neurotoxic influence of SOD1^G93A^ astrocytes in ALS models by regulating their mitochondrial respiration.

Here we provide evidence that DCA reduced astrocyte neurotoxicity to motor neurons in culture and furthermore, applied to ALS mice, slowed disease progression and enhanced motor strength.

## Results

### Effect of DCA on SOD1^G93A^ astrocytes

**Figure 2:**
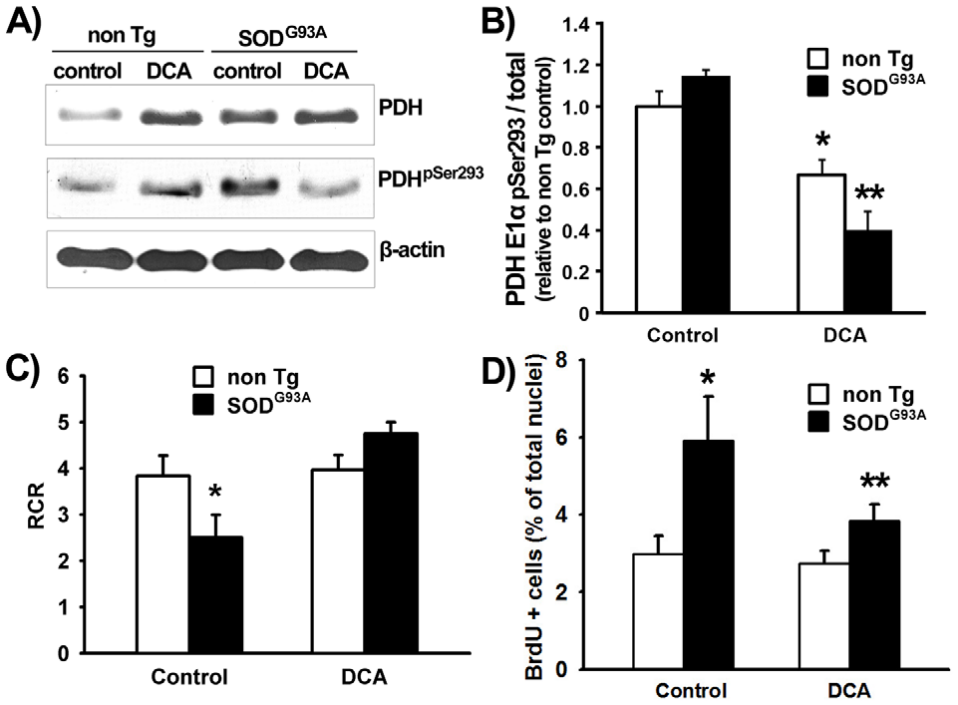
DCA recovers mitochondrial respiration rate and controls proliferation in SOD1^G93A^ astrocytes. A) Representative immunoblot for PDH-E1α(pSer^293^), total PDH-E1α, and β-actin of lysates from non Tg and SOD1^G93A^ astrocytes after 24 h treatment with DCA or vehicle as described in Methods. B) Quantification of the PDH-E1α(pSer^293^) to total PDH-E1α ratio between relative densitometric levels normalized against vehicle-treated non Tg astrocytes. C) Calculated respiratory control ratio (RCR) for mitochondria from non Tg or SOD1^G93A^-bearing astrocytes treated with DCA or vehicle as indicated. D) Percentage of BrdU immunoreactive nuclei of non Tg and SOD1^G93A^ astrocytes after 24 h treatment with DCA. Data for panels B, C, and D are expressed as mean ± SEM from three independent experiments performed in duplicate. *p<0.05, significantly different from non Tg control. **p<0.05, significantly different from SOD1^G93A^ control.

To evaluate the effects of DCA on PDH we exposed cultures of SOD1^G93A^ astrocytes to DCA (5 mM, 24 h) and total and phosphorylated forms of PDH were quantified by western blotting assay using specific antibodies (anti PDH-E1α subunit and anti PDH-E1α-pSer^293^ respectively). As expected, exposure of both non-transgenic (non Tg) and SOD1^G93A^ astrocytes to DCA reduced phosphorylated PDH relative levels (Fig. 2A–B). However, this effect was greater in SOD1^G93A^ astrocytes than in non Tg ones. DCA also increased total levels of PDH in non Tg astrocytes according to previous reports [14]. Total PDH levels were basally increased in SOD1^G93A^ untreated astrocytes.

DCA treatment improved mitochondrial coupling in SOD1^G93A^ astrocytes as determined by high-resolution respirometry expressed by the respiratory control ratio (RCR). The RCR value calculated for untreated SOD1^G93A^ astrocytes was significantly reduced (by 45%) compared to non Tg astrocytes as described previously [5]. DCA-treated SOD1^G93A^ astrocytes showed a significant increase in the RCR to the level of that shown by non Tg astrocytes (Fig. 2C). We observed that DCA also reduced the proliferation rate in SOD1^G93A^ astrocytes that was otherwise increased by 100% with respect to non Tg ones (Fig. 2D).

### DCA prevented the toxicity of SOD1^G93A^ astrocytes to motor neurons

**Figure 3:**
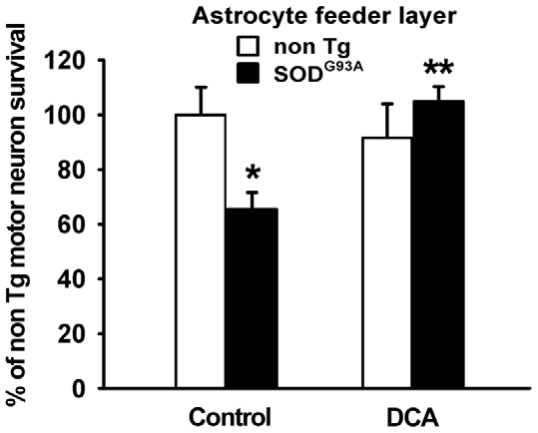
DCA prevents SOD1^G93A^ astrocyte neurotoxicity to motor neurons. Motor neuron survival 72 h after plating either on non Tg or SOD1^G93A^-bearing astrocytes pretreated with DCA or vehicle as indicated. Data are expressed as percentage of non Tg control, mean ± SEM from four independent experiments. *p<0.05, significantly different from non Tg control. **p<0.05, significantly different from SOD1^G93A^ control.

SOD1^G93A^ astrocytes display neurotoxic influence for motor neurons in culture, which can be reverted by mitochondria-targeted antioxidants [5]. In order to elucidate whether improving mitochondrial function with DCA in SOD1^G93A^ astrocytes was also beneficial for motor neuron survival we plated purified motor neurons on top of DCA-pretreated (0.5–5 mM, 24 h) SOD1^G93A^ astrocyte monolayers. This treatment significantly increased motor neuron survival grown on top of SOD1^G93A^ astrocytes to the level of that shown by non Tg astrocytes (Fig. 3).

### DCA increased survival of SOD1^G93A^ mice

**Figure 4:**
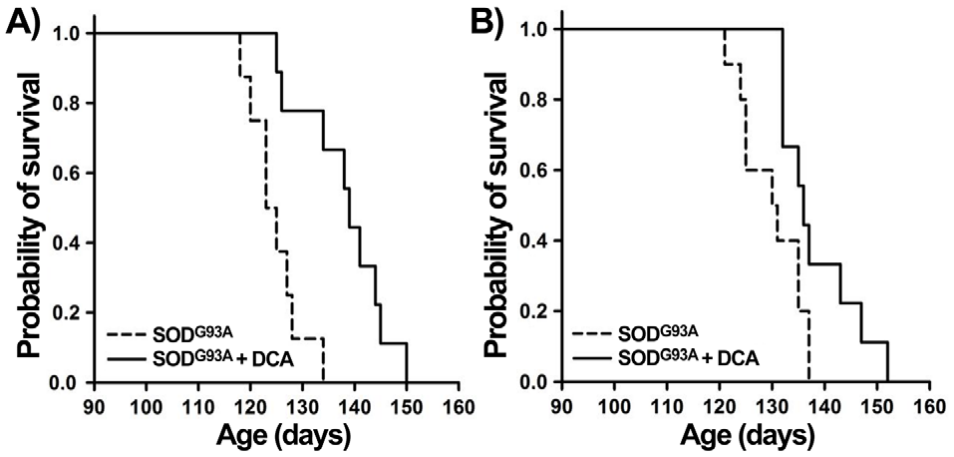
DCA increases mean survival of SOD1^G93A^ transgenic mice. Kaplan-Meyer survival curves from DCA-treated and control SOD1^G93A^ male (A) and female (B) mice. DCA was administered in drinking water from 70 days of age until death as detailed in materials and methods. 9 animals per group, p<0.05, Kaplan-Meyer log-rank test.

To assess whether DCA could also exert protective effects on the progressive paralysis in SOD1^G93A^ mice, the compound was administered from 70 days of age until death in the drinking water (500 mg/L) as previously described in an animal model of Huntington's disease [19]. DCA was well tolerated and did not show apparent signs of intoxication, such as weight loss, disease or premature death, when compared to non-treated SOD1^G93A^ and non Tg control mice. Treatment with DCA significantly increased survival both in males and females as compared with control mice treated with water only (males ctrl n = 9: 126.9±2.6 days, DCA n = 9: 138.0±2.8 days; females ctrl n = 10: 130.0±1.87 days DCA n = 9: 138.4±2.42 days; Fig. 4A, B). Disease onset was not significantly affected by DCA (males ctrl n = 7: 99.4±3.0 days; DCA n = 6: 106.2±2.3 days; females ctrl n = 5: 104.2 days±5.1; DCA n = 8: 111.3±6.7 days).

### DCA improved motor performance and maintained motor unit integrity in SOD1^G93A^ mice

**Figure 5:**
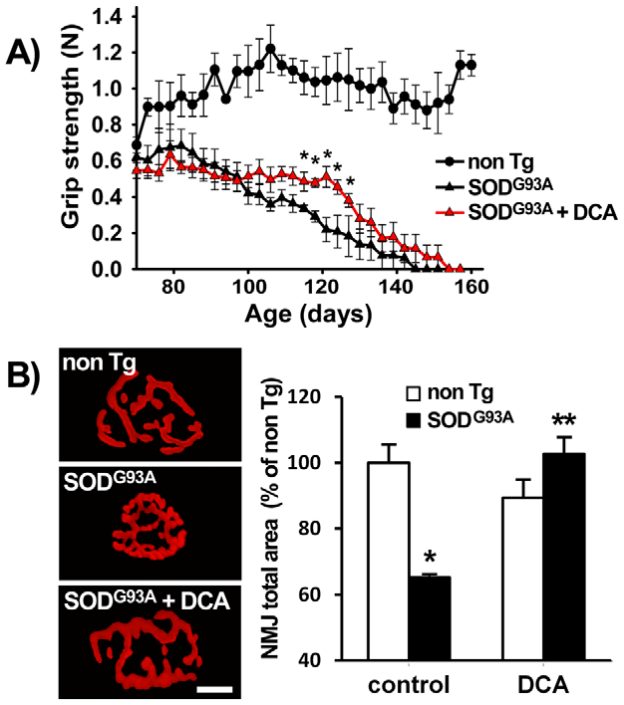
DCA delays loss of grip strength and neuromuscular junction shrinkage in SOD1^G93A^ mice. A) Hind-limb grip strength records from non Tg or SOD1^G93A^ male mice treated with DCA or vehicle as indicated. DCA-treated non Tg animals did not show differences with control ones and data are not shown in order to simplify the graph. Data are mean ± SEM from 9 animals per group. *p<0.05, significantly different from SOD1^G93A^ control. B) ACh receptors labeled with TMR-BgTx in representative EDL neuromuscular junctions from non Tg (top), SOD1^G93A^ control (middle) or DCA-treated SOD1^G93A^ (bottom). Quantification of total TMR-BgTx-stained neuromuscular area in the different groups of animals. Data are expressed as percentage of non Tg control, mean ± SEM from 15–35 neuromuscular junctions from 2–4 animals per group. *p<0.05, significantly different from non Tg control. **p<0.05, significantly different from SOD1^G93A^ control. Scale bar: 30 µm.

ALS is characterized by progressive muscle weakness and paralysis. Therefore, we sought to determine the effect of DCA on motor performance of SOD1^G93A^ mice. Notably, DCA significantly improved grip strength performance at the end stage of the disease (from 100 days of age onward) in male mice compared to control transgenic ones (Fig. 5A). Grip strength did not improve in females (data not shown). Because there is a correlation between the number of active motor units and the force produced by a muscle, we decided to observe the neuromuscular junction (NMJ) morphology of two muscles with different fiber composition, extensor digitorum longus (EDL) and soleus. The NMJs of EDL muscle in DCA-treated mice displayed normal size (measured as total area) and shape, which is in agreement with the improvement of grip strength (Fig. 5B). In control SOD1^G93A^ mice, the receptor area decreased in size, as did the spaces between synaptic regions, leading to an overall compaction of the junction. A 20-day DCA treatment from 70 days of age onward showed a significant increase in junction area in EDL muscles compared with untreated mice. Neuromuscular junctions in soleus muscles did not show significant improvements with DCA treatment (data not shown).

### DCA treatment improved mitochondrial function in the spinal cord of SOD1^G93A^ mice

**Figure 6:**
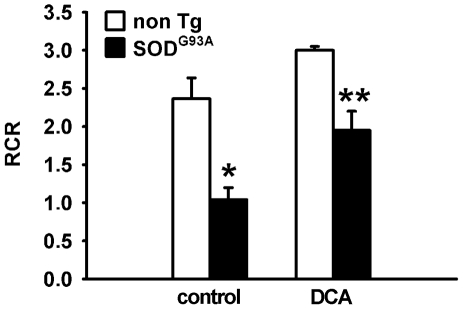
DCA improves mitochondrial function in the spinal cord of SOD1^G93A^ mice. Calculated respiratory control ratio (RCR) for spinal cord mitochondria from non Tg or SOD1^G93A^ mice treated with DCA or vehicle as indicated. Data are mean ± SEM from three independent experiments. *p<0.05, significantly different from non Tg control. **p<0.05, significantly different from SOD1^G93A^ control.

To assess whether DCA improved mitochondrial function *in vivo*, high-resolution respirometry was monitored in mechanically dissociated spinal cords obtained from mice after 20 days of DCA administration (from 70 days of age). The RCR value calculated for untreated SOD1^G93A^ mice spinal cords was significantly reduced compared to non Tg littermates according to previous reports [20]. In contrast, DCA-treated SOD1^G93A^ mice showed a significant increase in the RCR compared to the untreated group (Fig. 6).

### DCA reduced motor neuron loss and astrocyte reactivity in the spinal cord of SOD1^G93A^ mice

**Figure 7:**
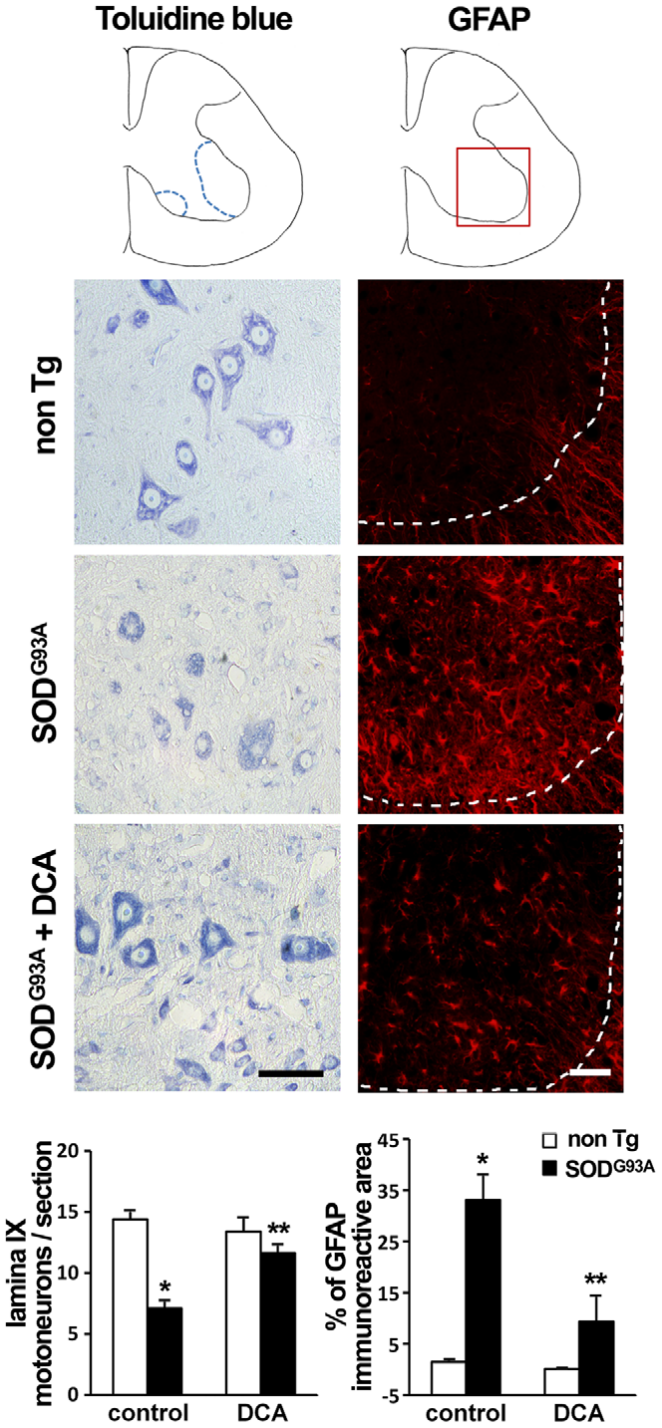
DCA reduces motor neuron loss and astrocyte reactivity in the spinal cord of SOD1^G93A^ mice. Representative Toluidine blue stain (left column) and GFAP immunofluorescence (red, right column) in anterior horn spinal cord sections from non Tg (top), SOD1^G93A^ control (middle) or DCA-treated SOD1^G93A^ (bottom) mice. Dotted lines in right column panels indicate the limit between grey and white matter. The graphs indicate the number of neuronal somas located in Rexed lamina IX (left) and the percentage of GFAP immunoreactive area in the ventral horn (right) in the indicated groups of animals. The corresponding measurement areas are drawn in the top. Data are mean ± SEM from at least three animals per group as indicated in Methods. *p<0.05, significantly different from non Tg control, **p<0.05, significantly different from SOD1^G93A^ control. Scale bars: 50 µm.

We sought to determine the effect of DCA administration in the loss of motor neurons in the spinal cord. We noted the already described reduced motor neuron somas at lumbar spinal segments for the SOD1^G93A^ mice when compared to non Tg littermates (Fig. 7 left column). DCA treatment rescued 25% of motor neurons at the lumbar level (Rexed lamina IX). In addition, we assayed astrocytic GFAP immunoreactivity in the spinal cord as a cellular element contributing to disease progression [8]. Astrocyte reactivity as determined by GFAP immunofluorescence was increased in the SOD1^G93A^ mice in contrast to non Tg littermates as previously described [21], [22]. DCA treatment induced a marked reduction (70%) in GFAP immunoreactivity in the spinal cord, when compared to the vehicle-treated group (Fig. 7 right column).

## Discussion

It is intriguing that rat [4], mouse [10], [11] and also human [23] astrocytes expressing mutated SOD1 exert toxic effects on motor neurons. Here, we report that DCA was sufficient to reverse mitochondrial dysfunction in astrocytes from SOD1^G93A^ rats and, at the same time, the phenotypic features that make astrocytes toxic for motor neurons. Significantly, we provide convincing preclinical data that systemic DCA administration decreases astrocytosis, motor neuron death and prolongs motor performance and survival of SOD1^G93A^ ALS mice. Since DCA has been used in humans for decades in the treatment of lactic acidosis and inherited mitochondrial diseases [18], these results suggest that DCA may be employed in clinical trials for ALS.

How DCA attenuates the damage induced by the expression of mutated SOD1 in astrocytes still needs to be clarified. Mitochondria represent a specific site of mutated SOD1 accumulation. In particular, it accumulates in the outer membrane and the intermembrane space, where it induces mitochondrial damage and metabolic dysfunction [3], [5]. Oxidative stress to astrocyte mitochondria may underlie the transformation to a neurotoxic phenotype [5], [24]. Reactive oxygen species (ROS) produced in mitochondria may affect signal transduction pathways through oxidation/reduction of cysteine residues in kinases, phosphatases, and other regulatory factors [25] leading to different, even opposite cellular responses. By stimulating pyruvate consumption, DCA improves the redox balance of mitochondria, which may result in normalization of altered mitochondria-regulated signaling [26]. Furthermore, DCA applied to mice at doses similar to the ones we have used but for a longer period of time significantly induced antioxidant enzyme activities, including superoxide dismutase, catalase and glutathione peroxidase [27], suggesting an indirect antioxidant effect of DCA.

Interestingly, DCA beneficial effects on survival and motor performance were more pronounced in males than in females. Similar results have been described in previous studies where different approaches resulted in beneficial effects only in males [28]. Men have higher risk of suffering ALS than women [29], and sex differences have also been reported in SOD1^G93A^ mutant rats [30] and mice [31]–[34] particularly when large numbers of animals are compared [35]. These observations suggest that gender differences may be partially a result of sex hormone action and in fact estrogen modulates disease progression in SOD1^G93A^ mice [36], [37]. Among the various neuroprotective mechanisms proposed for estrogen actions, the ability to modulate mitochondrial function [38] and oxidative stress is particularly interesting in this context. Furthermore, the fact that increased ROS levels were observed in SOD1^G93A^ males but not in females [28] suggests that in the latter the redox balance between pro- and anti-oxidant mechanisms is already shifted toward neuroprotection, masking DCA beneficial effects.

Previous works have suggested an alteration of mitochondrial redox metabolism in ALS. Indeed, recent metabolomic analysis of cerebrospinal fluid [39] or serum [40] by ^(1)^H NMR spectroscopy in ALS patients, revealed abnormal metabolite patterns that may indicate perturbation of glucose metabolism. Among the approaches that aim to restore mitochondrial function and energy production, pyruvate administration to ALS mice has been assessed [41], [42]. However, results were contradictory probably due to differences in doses and animal strains. Nonetheless, pharmacological administration of DCA did improve mitochondrial function underscoring the potential of metabolic modulation to neutralize the progression of the disease.

In addition to mitochondrial dysfunction, SOD1^G93A^ astrocytes also exhibited an increased proliferation rate. Although these two events might seem unconnected, evidence obtained in cancer cells indicates that in fact they are mutually related. Otto Warburg in the 1920s described that increased proliferation rate in cancer cells is associated to glycolytic metabolism rather than to mitochondrial oxidation of pyruvate [43]. In accordance, DCA reverses the glycolytic metabolism in several cancer cell lines along with reduced proliferation [44]. Our data showing that DCA treatment of SOD1^G93A^ astrocytes improved mitochondrial function and also decreased their proliferation rate suggest common activation of transduction pathways between cancer cells and SOD1^G93A^ astrocytes. Moreover, we have found evidence of unregulated proliferation and lack of replicative senescence in a population of phenotypically aberrant astrocytes isolated from SOD1^G93A^ rats [12]. Thus, the possibility exists that chronic mitochondrial dysfunction in neonatal astrocytes promotes long term changes in astrocyte phenotype and their neurotoxic potential. Although not analyzed in our study, several cellular pathways might mediate the increased proliferation rate of SOD1^G93A^ astrocytes. Among them we can include up-regulation of the isoform A of the lactate dehydrogenase (LDH-A) [45], activation of E3 ubiquitin ligase APC/C-Cdh1 [46], or modulation of transduction pathways by depolarized mitochondria [47].

Systemic DCA likely improves mitochondrial status in several other cell types relevant to ALS, including neurons and skeletal muscle. In the central nervous system, astrocytes seem to preferentially respond to DCA due to having a greater fraction of total PDH in the inactive phosphorylated form than neurons [15]. DCA might also exert a direct effect on skeletal muscle. It is interesting to note that DCA-treated animals remained with increased grip strength until death compared to untreated animals. Noticeably, the EDL muscle fibers of DCA-treated mice displayed neuromuscular junctions with normal size and shape, suggesting DCA could delay the progressive neuromuscular junction destruction characteristic of animals with ALS [48]. Furthermore, DCA has been effective in recovering the function of ischemic muscle [49], suggesting an effect stimulating muscle trophism or decreasing deleterious inflammation.

The present study supports the potential use of DCA in multi-drug approaches to treat ALS. DCA has been also shown to be protective in models of Huntington's disease, which also involves non-cell-autonomous mechanisms [19], suggesting a more general effect in neurodegenerative diseases. Taken together these data raise the possibility that DCA might have therapeutic benefits in ALS patients.

## Materials and Methods

### Materials

Culture media and serum were obtained from Invitrogen (Carlsbad, CA). All other reagents were from Sigma Chemical Co (Saint Louis, MO) unless otherwise specified.

### Ethics Statement

Procedures using laboratory animals were in accordance with international guidelines and were approved by the Institutional Animal Committee (Comisión honoraria de experimentación animal de la Universidad de la República http://www.chea.csic.edu.uy; CHEA).

### Animals

Transgenic ALS mice carrying the G93A mutation for human SOD1, strain B6SJL-TgN(SOD1-G93A)1Gur [50], were obtained from Jackson Laboratories (Bar Harbor, ME, USA) and genotyped as previously described [51]. Mice were housed under controlled conditions with free access to food and water. Sprague-Dawley NTac:SD-TgN(SOD1G93A)L26H rats were obtained from Taconic (Hudson, NY; [52]) and were bred locally by crossing with wild-type Sprague-Dawley female rats.

### Dichloroacetate treatment trial

Male and female transgenic mice and non-transgenic littermates were divided randomly into the following groups (n = 9 per group): A) transgenic control and B) non-transgenic control groups which received regular drinking water; C) transgenic DCA treatment and D) non-transgenic DCA treatment groups, administered with dichloroacetate (DCA; Sigma, St. Louis, MO). DCA was added to tap water in a 500 mg/L concentration and placed into water bottles. A daily dose of 100 mg/kg was used based on a daily water intake of 5 ml. The DCA solution was made fresh twice a week, with the total consumed volume measured in order to ensure a constant dose.

The treatment was performed from presymptomatic stage (70 days old) to death. Animals were observed weekly for onset of disease symptoms, as well as progression to death. Onset of disease was scored as the first observation of abnormal gait or overt hind limb weakness. End-stage of the disease was scored as complete paralysis of both hind limbs and the inability of the animals to right themselves after being placed on their side.

### Cell cultures

Astrocyte cultures: Primary rat spinal cord astrocyte cultures were prepared from transgenic SOD1^G93A^ and non-transgenic 1-day-old pups, genotyped by PCR, as previously described [4], [53]. Briefly, cells were plated at a density of 2×10^4^ cells/cm^2^ in 35 mm Petri dishes or 24-well plates (Nunc, Naperville, IL, USA) and maintained in Dulbecco's modified Eagle's medium (DMEM) supplemented with 10% fetal bovine serum (FBS), HEPES (3.6 g/L), penicillin (100 IU/mL) and streptomycin (100 µg/mL). Astrocyte monolayers were >98% pure as determined by GFAP immunoreactivity.

Astrocyte-motor neuron cocultures: motor neuron preparations were obtained from embryonic day 15 (E15) rat spinal cord by a combination of optiprep (1∶10 in L15 medium, SIGMA St. Louis, MO) gradient centrifugation and immunopanning with the monoclonal antibody IgG192 against p75 neurotrophin receptor as previously described [53], then plated on rat astrocyte monolayers at a density of 300 cells/cm^2^ and maintained for 48 h in L15 supplemented medium as described [53].

### Treatment of cultures and motor neuron counting

Confluent astrocyte monolayers were changed to L15 supplemented media prior treatment. Stock solution of DCA (SIGMA St. Louis, MO) was prepared in distilled water and directly applied to astrocyte monolayers at the indicated concentrations. For co-culture experiments, astrocyte monolayers were treated with DCA for 24 h and motor neurons were plated in fresh L15 supplemented media, after washing twice with Dulbecco's phosphate buffered saline (DPBS). Motor neuron survival was assessed after 48 h by directly counting all p75 immunoreactive cells displaying neurites longer than 4 cell bodies in diameter [53].

### Proliferation assessment

Confluent astrocyte cultures were incubated with 10 µg/ml bromo deoxyuridine (BrdU) and 5 mM DCA or vehicle in DMEM 2% FBS for 24 h. Cells were fixed in 4% paraformaldehyde, incubated in 1 N HCl for 1 h and immunofluorescence to detect BrdU using 1∶300 diluted mouse monoclonal antibody (Dako) was performed. Alexa Fluor^488^ conjugated goat anti-mouse (Invitrogen) was used as secondary antibody, and propidium iodide to counterstain total nuclei. At least 600 nuclei were counted for each point. Proliferation is expressed as the percentage of BrdU immunoreactive nuclei with respect to total propidium iodide stained nuclei.

### Immunoblot analysis

Cells were treated with DCA as described above. After 24 h of treatment, proteins were extracted from cells in 1% SDS supplemented with 2 mM sodium orthovanadate and Cømplete protease inhibitor cocktail (Roche). Lysates were resolved by electrophoresis on 12% SDS-polyacrylamide gels and transferred to a polyvinylidene fluoride membrane (PVDF; Thermo). The membrane was blocked for 1 h at room temperature in 5% skimmed milk in TBS-T (Tris-buffered saline with 0.1% Tween). The membrane was then probed overnight with primary antibodies in 1% skimmed milk in TBS-T at 4°C, washed in TBS-T, and then probed with the appropriate horseradish peroxidase (HRP)-conjugated secondary antibody for 60 min at room temperature. Primary antibodies were rabbit polyclonal phosphodetect anti-PDH-E1α(pSer^293^) 1∶500 (AP1062; Calbiochem) and mouse monoclonal anti-PDHE1α 1∶750 (#456600, Invitrogen). Secondary antibodies were anti-rabbit (1∶2500) and anti-mouse (1∶5000) HRP conjugates (Thermo). Proteins were visualized with ECL western blotting substrate (Pierce Biotechnology). β-actin was used as a loading control. Densitometric analysis was performed using ImageJ software. The relative levels of pSer^293^ E1α and total E1α were quantified. The pSer^293^ to total E1α ratio was calculated and normalized against vehicle-treated non Tg mice.

### Histological analysis and immunofluorescence

Transgenic and non-transgenic mice groups (n = 3 per group) were treated as described above, from 70 days of age. After 20 days, mice were transcardially perfused with 4% paraformaldehyde fixative in DPBS under deep anesthesia (pentobarbital, 50 mg/kg i.p.). The lumbar spinal cords were postfixed and embedded in paraplast. 5 µm-thick, serial sections were stained with toluidine blue or processed for immunofluorescence. For GFAP immunodetection, sections were permeabilized (0.2% Triton X-100 in PBS) and unspecific binding blocked (10% goat serum, 2% BSA, 0.2% Triton X-100 in DPBS), incubated with primary antibody (mouse monoclonal Cy-3 conjugated anti-GFAP; 1∶600, Sigma) overnight, and mounted with glycerol. Images were obtained using an Olympus IX81 epifluorescence microscope.

### Assessment of motor neuron number and astrogliosis in the lumbar spinal cord

The number of motor neurons was assessed by counting every cell on lamina IX of Rexed displaying motor neuron morphology with nucleus and nucleolus on every fifth toluidine blue stained 5 µm section (at least 25 sections per animal) through the lumbar spinal cord.

Quantification of astrogliosis was performed on images obtained from every fifth GFAP immunostained section (20 sections from each group) using ImageJ software (NIH). Ventral horn area occupied by GFAP immunofluorescence was measured and expressed as a percentage of total ventral horn area in each section.

### Oxygen consumption

Oxygen consumption studies were performed either on spinal cord tissue or in astrocyte monolayers. Tissue or cell respiration was evaluated using Oxygraph 2 K (Oroboros Instruments Corp). Oxygen consumption was recorded at 37°C in intact cells or spinal cord tissue. The rate of oxygen consumption was calculated by means of the equipment software (DataLab) and was expressed as pmol of O_2_·s^−1^·ml^−1^.

For cell respiration, astrocyte monolayers were treated with DCA (0.5 and 5 mM) or vehicle for 24 h. Then, astrocytes were scraped and resuspended at 2×10^6^ cells/ml in culture medium. Mitochondrial oxygen consumption and RCR (respiratory control ratio) was calculated as: RCR = maximum uncoupled flux (FCCP)−(antimycin A-inhibited flux)/(oligomycin-inhibited flux)−(antimycin A-inhibited flux) of intact cells respiring, oxygen consumption after addition of 2 µg/ml oligomycin, 0.5 µM steps of FCCP (carbonyl cyanide p-trifluoromethoxyphenylhydrazone and followed by 2.5 µM antimycin A respectively as described [54].

For spinal cord studies 70 day-old mice were treated with DCA or vehicle for 20 days (n = 3 per group). Then, immediately following sacrifice, lumbar spinal cords were dissected. Spinal cord samples were immediately rinsed in respiration medium (Sucrose 110 mM, Mops 60 mM, EGTA 0.5 mM, BSA 1 g/l, MgCL2 3 mM, KH_2_PO_4_ 10 mM, HEPES 20 mM, pH 7,1) and set in the Oroboros oxygraph for high resolution respirometry. Mitochondrial oxygen consumption was measured as indicated in cell studies.

### Grip strength measurements

Motor function was tested with a Grip Strength Meter (San Diego Instruments, San Diego CA). Tests were performed by allowing the animals to grasp the platform with both hind limbs, followed by pulling the animals until they released the platform. The force measurement was recorded twice a week from week 6 (baseline) until death in four separate trials.

### Neuromuscular junction measurements

The extensor digitorum longus (EDL) and the soleus muscles from the same animals used for oxygen consumption studies were dissected immediately after sacrifice. Muscles were immersed for 60 minutes in 0.5% paraformaldehyde in DPBS, rinsed with DPBS (three times, 15 minutes each) and mechanically dissociated into small bundles of fibers using fine forceps under a stereomicroscope. The teased fibers were incubated with a blocking buffer containing 50 mM glycine, 1% BSA and 0.5% Triton X-100 for at least 3 hours and then in tetramethylrhodamine-conjugated α-bungarotoxin (TMR-BgTx) (T0195 Sigma; 1∶1500 in blocking buffer) overnight (ON) at 4°C. After washing (three times, 20 minutes each) under agitation with PBS, fibers were left ON at 4°C in glycerol-Tris pH 8.8 (4∶1) which was used as mounting medium for all the preparations.

Image acquisition and quantitative image processing: the teased fiber preparations were observed by epifluorescence using an Olympus IX81 microscope. For each EDL muscle the images of 15–35 neuromuscular junctions were taken and stored for later analysis using Adobe Photoshop software. The total area, defined as the area delimited by the external outline of the TMR-BgTx -stained endplate marked with the Lasso tool, including both stained and non-stained areas was measured. The resulting numbers of selected pixels were counted with the Histogram tool. Data were expressed as percentage of neuromuscular junction total area from non transgenic control animals.

### Statistics

Survival curves were compared by Kaplan-Meier analysis with the Log-rank test using Sigmaplot 12 (Systat software). All culture assays were performed in duplicate and each experiment was repeated at least three times. Quantitative data were expressed as mean ± SEM and ANOVA and Student's t test were used for statistical analysis, with p<0.05 considered significant. When the normality test failed, comparison of the means was performed by one-way ANOVA on ranks followed by the Kruskal–Wallis test. Data from GFAP were analyzed using a one-way ANOVA and compared by all pairwise multiple comparison procedures (Holm-Sidak method). All statistics computations were performed using the Sigma Stat System (1994, Jandel Scientific, San Rafael, CA, USA), or GraphPad InStat software, version 3.06.
